# Influence of Zinc Chloride Exposure on Microstructure and Mechanical Behavior of Age-Hardened AZ91 Magnesium Alloy

**DOI:** 10.3390/ma17184474

**Published:** 2024-09-12

**Authors:** Pavel Konopík, Tomasz Bucki, Sylwia Rzepa, Daniel Melzer, Dana Bolibruchová, Ying Li, Jan Džugan

**Affiliations:** 1 COMTES FHT a.s., Průmyslová 995, 334 41 Dobřany, Czech Republic; pavel.konopik@comtesfht.cz (P.K.); melzer.daniel@seznam.cz (D.M.); jan.dzugan@comtesfht.cz (J.D.); 2 Department of Metal Science and Materials Technology, Kielce University of Technology, Al. Tysiąclecia Państwa Polskiego 7, 25-314 Kielce, Poland; tbucki@tu.kielce.pl; 3 Academic Centre for Materials and Nanotechnology, AGH University of Krakow, Mickiewicza 30, 30-059 Kraków, Poland; srzepa@agh.edu.pl; 4 Department of Technological Engineering, University of Zilina, Univerzitná 8215/1, 010 26 Žilina, Slovakia; danka.bolibruchova@fstroj.uniza.sk

**Keywords:** magnesium alloys, coatings, thermochemical treatment, age hardening, electron microscopy, stress/strain measurements

## Abstract

The AZ91 magnesium alloy was subjected to a complex treatment involving age hardening (supersaturation and artificial aging) and simultaneous surface layer modification. The specimens were supersaturated in contact with a mixture containing varying concentrations of zinc chloride, followed by cooling either in air or water. After supersaturation, the specimens were subjected to artificial aging and then air-cooled. This process resulted in the formation of a surface layer made of zinc-rich phases. The thickness and microstructure of the surface layer were influenced by the process parameters, namely, the zinc chloride content in the mixture and the cooling rate during supersaturation. The treated specimens exhibited favorable tensile strength and greater elongation compared to the as-cast AZ91 alloy, with values comparable to those of the alloy subjected to standard T6 tempering. No cracking of the layer was observed under moderate deformation, though greater deformation resulted in the formation of cracks, primarily in the areas containing the Mg_5_Al_2_Zn_2_ intermetallic phase. The produced layer demonstrated strong metallurgical bonding to the AZ91 substrate.

## 1. Introduction

The utilization of magnesium alloys offers a significant reduction in the weight of structures across various industrial sectors, including ground transportation, aerospace, and electronics. Nevertheless, the low resistance of these materials to corrosion and abrasion poses substantial obstacles that restrict their application [1,2]. In recent years, extensive research has shown that the properties and applicability of Mg-based materials can be noticeably enhanced through surface treatment, such as weld surfacing processes [3,4,5,6], cold spraying [7], thermochemical treatment [8,9,10,11,12,13,14,15,16], electroless deposition [17], and casting processes [18,19]. The literature indicates that an effective approach is to enrich the surface of Mg alloys with elements that form hard intermetallic phases with magnesium, such as Al [3,4,5,9,10,11,12,18], Zn [7,8,14,15,16], Cu [3], Ni [3,6], Si [3,19], Al + Cu [3], Al + Ni [3], and Al + Si [3,5,13]. Among these techniques, thermochemical treatment stands out due to its simplicity and low manufacturing cost. The process involves heating the specimen in contact with a source of diffusing atoms, which can be elements or compounds in various states under the process conditions. Popular solid media include powders, such as Al powder [9], which diffuse into the Mg alloy, typically resulting in the formation of a surface layer containing Mg-Al intermetallic phases. Thermochemical treatment in a liquid medium can be carried out using molten salts, such as a mixture of AlCl_3_ and NaCl [10,11,12]. In this scenario, a layer composed of Mg-Al phases is formed as a result of the reaction of AlCl_3_ with the alloy surface and simultaneous diffusion processes. The resulting surface layers have been reported to act as effective corrosion barriers, significantly increasing the corrosion resistance of the magnesium alloy. Surface treatment of magnesium or magnesium alloys can also be performed by heating products coated with a ZnCl_2_-containing paste. The method described by Bucki et al. [14,15] involves heating Mg-based specimens in contact with a source of Zn atoms in the form of a paste containing ZnCl_2_ mixed with a filler (KCl + pine rosin + ethanol). This process results in the formation of a surface layer containing Mg-Zn intermetallic phases due to the reaction between the Mg substrate and the Zn compound. The literature on Zn enrichment using various techniques also indicates the potential for improving the corrosion resistance of magnesium alloys [7,8]. However, the properties of the Zn-enriched surface layer produced by thermochemical treatment seem to be insufficiently studied in the available literature. This study focuses on exploring this method and analyzing the microstructures and properties of the produced surface layer.

Based on preliminary tests, it was found that the mixture for coating of Mg-based alloys can also be prepared by mixing ZnCl_2_ with CaSO_4_·0.5H_2_O (plaster of Paris) filler with the addition of water. This novel coating mixture was applied in the present study because it allows for the control of the structure and properties of the produced layer by selecting the proportions of the components and the process parameters. The aim of this study was to analyze the phase composition of the surface layers produced at different process parameters. Moreover, since age hardening can occur during the process, the study also focused on this aspect, with mechanical property assessments, supplemented by fractographic observations.

## 2. Materials and Methods

The material used in the study was an ingot of magnesium AZ91 cast alloy with the following composition: 9.14 wt.% Al, 0.64 wt.% Zn, 0.23 wt.% Mn, and Mg-balance. The ingot was cut into specimens with dimensions of 40 × 10 × 2 mm^3^. The specimens were next ground with abrasive paper up to 800 grit and cleaned in ethanol. The complex treatment included heating the AZ91 specimen in contact with a source of Zn atoms in the form of zinc chloride powder that was mixed with a filler (plaster of Paris powder). The substances of technical grade were used, i.e., min. 98 wt.% ZnCl_2_ and min. 95 wt.% CaSO_4_·0.5 H_2_O, respectively). In order to determine the optimal composition, different compositions of the ingredients were applied (content of ZnCl_2_: 3, 4, or 5 in wt.%). The powders were next mixed with sufficient water to obtain a thick consistency. Then, the surfaces of the specimens were covered with the obtained mixture to a height of approx. 10 mm above the top surface of the specimen. After cementation of the covering medium, the specimens were heated in a chamber furnace to modify the surface layer with simultaneous supersaturation of the AZ91 substrate. The age hardening parameters were adopted from previous studies [20,21]. Supersaturation took place for 24 h at a temperature of 425 °C without any protective atmosphere. After removal from the furnace, the specimens with the covering medium were cooled in air (AC) or water (WC). The next step of the process was the artificial aging of the specimens for 16 h at 175 °C without the use of a protective atmosphere, followed by air cooling. For comparative purposes, specimens subjected to age hardening without contact with covering medium were also produced (standard T6 temper). In this variant, the same heat treatment parameters were used, and the supersaturated specimens were also cooled in air or water. However, an argon atmosphere was used to protect the Mg-based alloy from oxidation during heating. The variants of the process parameters are summarized in Table 1. For clearly describing the work, the variants are marked with symbols.

The microstructure of the modified surface layers was observed in the central part of the upper surface of the specimens using a Nikon ECLIPSE MA 200 optical microscope (OM) (Nikon, Tokyo, Japan) and a JEOL JSM-7100F scanning electron microscope (JEOL Ltd., Tokyo, Japan) equipped with an energy-dispersive X-ray spectroscope detector (SEM/EDS). The metallographic specimens were prepared according to standard procedures. The final polishing was performed using colloidal silica. The microstructural components have been self-etched in contact with water. The measurement of the thickness of surface layers was conducted during OM observations for at least 3 regions on the observed cross-section of the specimen. X-ray diffraction (XRD) analysis was conducted with polished specimens, using Bruker Advance D8 diffractometer with a copper anode (λ Kα1 = 1.54056 nm) (Bruker AXS GmbH, Karlsruhe, Germany). The range of 2θ angle was from 30° to 90° with an increment of 0.025°.

**Figure 1:**
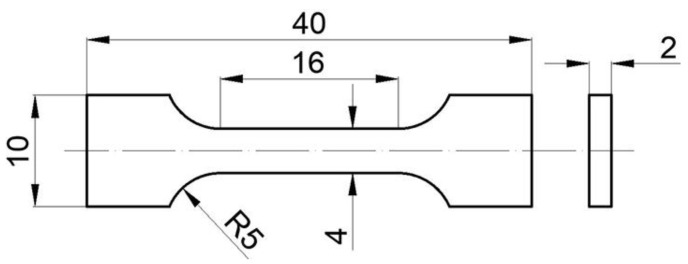
Geometry of tensile test specimen (in mm).

The mechanical properties of the materials produced, together with the behavior of their surface layers during deformation, were examined by uniaxial tensile test performed on an electromechanical universal testing machine Mayes (DM100, W.H. Mayes & SON, Windsor, UK) with a load cell capacity of 100 kN at room temperature. The tensile characteristics were determined according to ASTM E8/E8M [22] by testing at least 3 specimens with standard dimensions (as shown in Figure 1). The tensile test was controlled by constant crosshead velocity of the machine (0.2 mm/min). During the test, the deformation of the surface layer was tracked by two high-precision optical systems based on Digital Image Correlation (Aramis professional 2020, ZEISS, Oberkochen, Germany). The surfaces of the specimens to be tested were ground using 2500 grit abrasive paper. The surface recorded by the ARAMIS system was additionally painted in accordance with the technical requirements of the system. A graphical representation of the deformation of the surface layer recorded by the video recording system was hampered by subtle structural changes and the reflectivity of the specimens. Nonetheless, this analysis was intended to provide quantitative information on the fracture mechanism of the surface layer. The specimens after fracture were subjected to further fractographic observations.

## 3. Results

**Figure 2:**
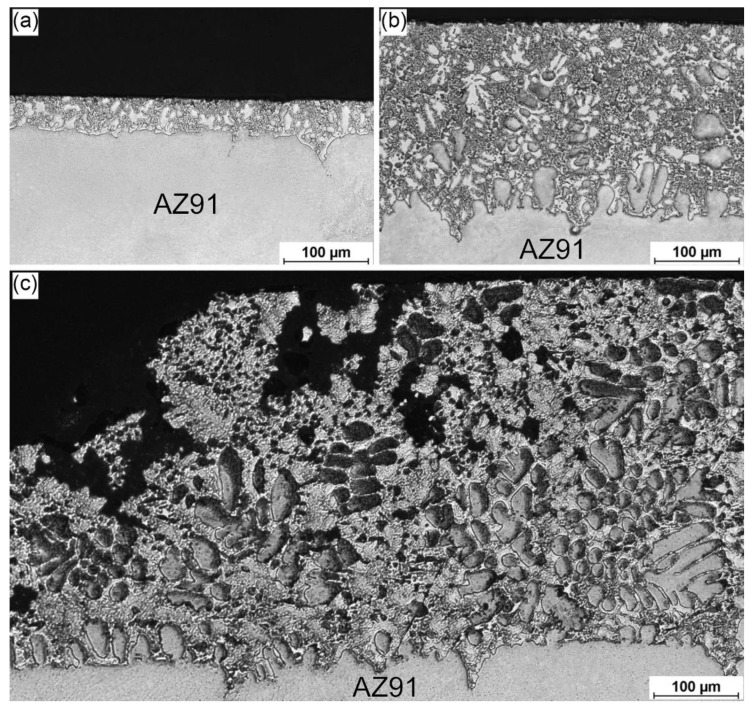
Microstructure of the surface layers observed in OM; treatment variants: (**a**) 3ZnCl_2__AC; (**b**) 4ZnCl_2__AC; and (**c**) 5ZnCl_2__AC.

Figure 2 shows the microstructure of specimens fabricated in contact with mixtures containing various contents of ZnCl_2_, which were air-cooled during supersaturation (designated as 3ZnCl_2__AC, 4ZnCl_2__AC, 5ZnCl_2__AC, respectively). The results revealed that the addition of ZnCl_2_ content resulted in the modification of the surface layer of the AZ91 alloy. For the 3ZnCl_2__AC variant (Figure 2a), the fabricated surface layer had a uniform and relatively small thickness of about 40 µm, with the layer partially penetrating the AZ91 substrate along the grain boundaries. The composition of the formed layer was complex; there were light dendrites distributed over the two-phase matrix. Increasing the ZnCl_2_ content to 4% (Figure 2b) led to the production of a surface layer with a clearly greater thickness, ranging from 200 to 240 µm. The layer exhibited a similar microstructure to that of the 3ZnCl_2__air variant but additionally included dark dendrites. The treatment with a mixture containing 5% ZnCl_2_ (Figure 2c) resulted in the formation of a layer with uneven thickness, varying from 100 to 440 µm. Porosity and irregularities, likely due to the flow of the liquid material, were observed locally in the specimen. The presented defects also contributed to macroscopic unevenness of the specimen surfaces.

**Figure 3:**
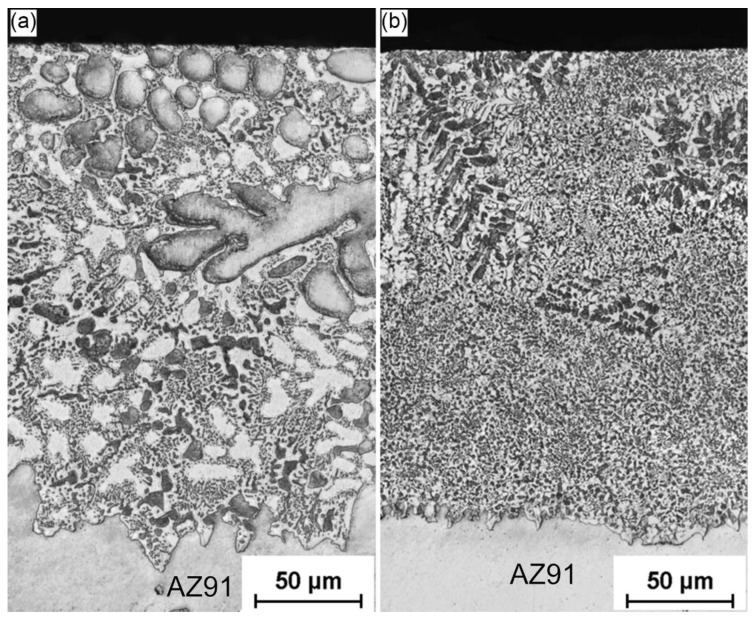
High-magnification images of the surface layers for variants: (**a**) 4ZnCl_2__AC and (**b**) 4ZnCl_2__WC.

Due to the relatively large and uniform thickness of the layer produced for the 4ZnCl_2__air variant, the treatment medium containing 4% ZnCl_2_ was selected for further tests. Figure 3 compares the microstructure details of the specimen cooled in air (4ZnCl_2__AC) with those subjected to water cooling (4ZnCl_2__WC) to analyze the effect of the cooling rate after supersaturation on the formation of the layer. The results clearly demonstrate that the cooling rate has a significant effect on the microstructure of the formed layer. The higher cooling rate in the 4ZnCl_2__water variant led to a great refinement of the surface layer constituents, resulting in fine particles of light and dark phases interspersed with slightly coarser gray dendrites.

**Figure 4:**
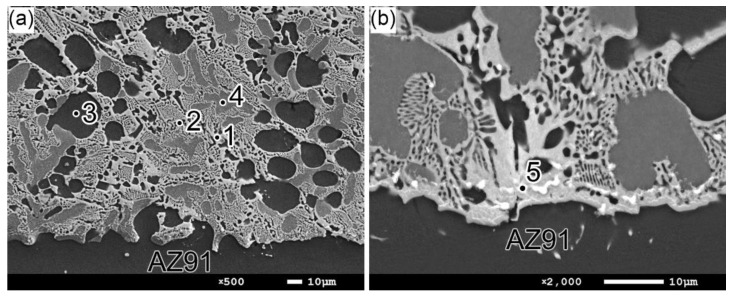
The SEM microstructure of the surface layer of the 4ZnCl_2__AC variant: (**a**) lower magnification and (**b**) higher magnification of the area close to the interface with the AZ91 substrate.

Figure 4a presents the SEM microstructure of the surface layer for the 4ZnCl_2__AC variant. The results of the EDS point analysis for the areas marked in this figure are listed in Table 2. Based on the literature on the Mg-Al-Zn phase equilibrium system [23,24], it was possible to determine the phase composition of the produced layer. The composition of the two-phase matrix of the layer (marked as points 1 and 2) indicates that this area is composed of a eutectic mixture containing an Mg-solid solution and an Mg_5_Al_2_Zn_2_ intermetallic phase. The high content of Mg in the area marked as point 3 suggests that the dark dendrites are composed of an Mg-solid solution. The chemical composition of the lighter dendrites (point 4) indicates the presence of the Mg_17_Al_12_ phase. The noticeable Zn content in this region corresponds to the findings of Ren et al. [24], which suggest that Al atoms in the Mg_17_Al_12_ phase can be replaced by Zn, resulting in the Mg_17_(Al,Zn)_12_ phase. Further SEM observations at higher magnification (Figure 4b) revealed that the surface layer also contains light particles in the form of threads or equiaxed shapes. They were concentrated mainly close to the interface with the AZ91 substrate in the background of the eutectic mixture. The analysis at point 5 indicates that these particles are composed of the MgZn phase. The effect of age hardening on the microstructure of the AZ91 substrate was not analyzed in detail, as it has been extensively covered in previous studies. For instance, Dziadoń et al. [20] showed that the microstructure of the AZ91 alloy after age hardening consists of an Mg-solid solution matrix with fine Mg_17_Al_12_ intermetallic phase particles in the form of continuous and discontinuous precipitates.

**Figure 5:**
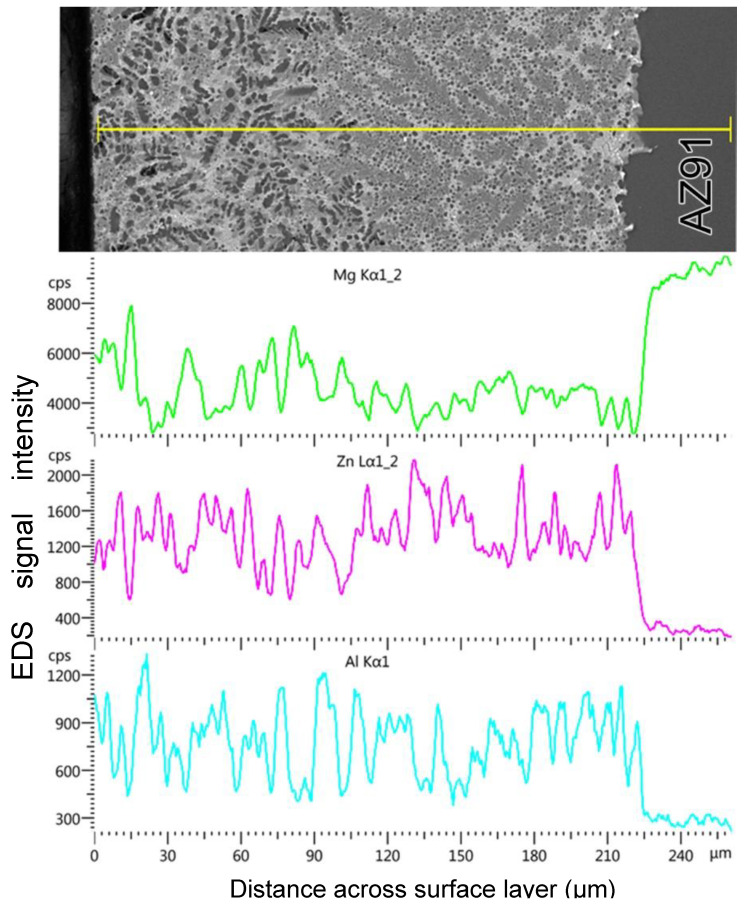
Linear EDS analysis across the surface layer for the 4ZnCl_2__WC variant with the corresponding distribution of Mg, Zn, and Al.

For the 4ZnCl_2__WC variant, the high dispersion of the structural components within the layer made it impossible to perform quantitative analyses by the EDS method. However, SEM observations together with EDS analysis and the distribution of Mg, Zn, and Al along the line marked in Figure 5 indicated a phase composition similar to the air-cooled variant.

**Figure 6:**
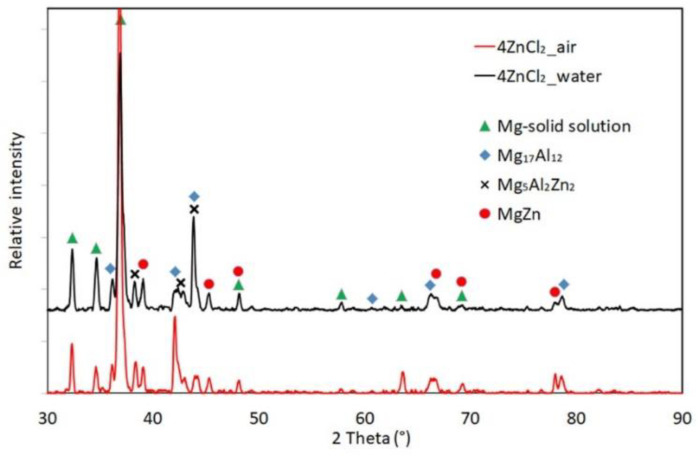
Results of the XRD analysis in the surface layers for specimens designated as 4ZnCl_2__AC and 4ZnCl_2__WC.

Figure 6 presents diffractograms of the XRD analyses performed in the surface layers for specimens designated 4ZnCl_2__AC and 4ZnCl_2__WC. The results confirmed the previous SEM/EDS findings, as they revealed that, in both cases, the layer was composed of the following phases: Mg_5_Al_2_Zn_2_, Mg-solid solution, Mg_17_Al_12_, and MgZn.

**Figure 7:**
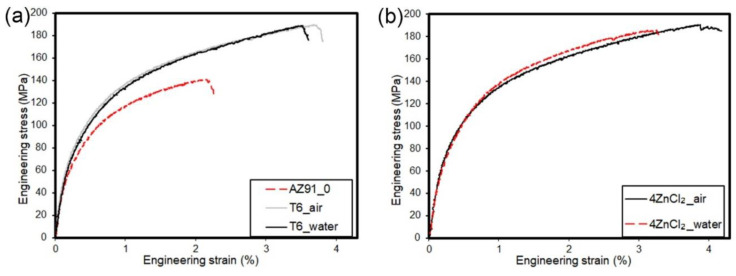
Representative tensile curves for the analyzed specimens: (**a**) uncoated specimens and (**b**) specimens with a surface layer.

Table 3 summarizes the results of the tensile test for the analyzed variants, while the representative tensile curves are presented in Figure 7. The results indicate that the as-cast AZ91 alloy exhibited relatively poor strength properties and low elongation compared to the processed specimens. Subjecting AZ91 to standard T6 age hardening, with both water and air cooling, led to a significant improvement in yield strength and tensile strength as well as an increase in elongation. The specimens that underwent simultaneous surface modification and age hardening (for both variants: 4ZnCl_2__AC and 4ZnCl_2__WC) showed comparable results to those achieved with standard T6 age hardening. However, the tensile curves for these variants revealed sudden drops at significant plastic deformation, likely due to the rupturing of the surface layer.

**Figure 8:**
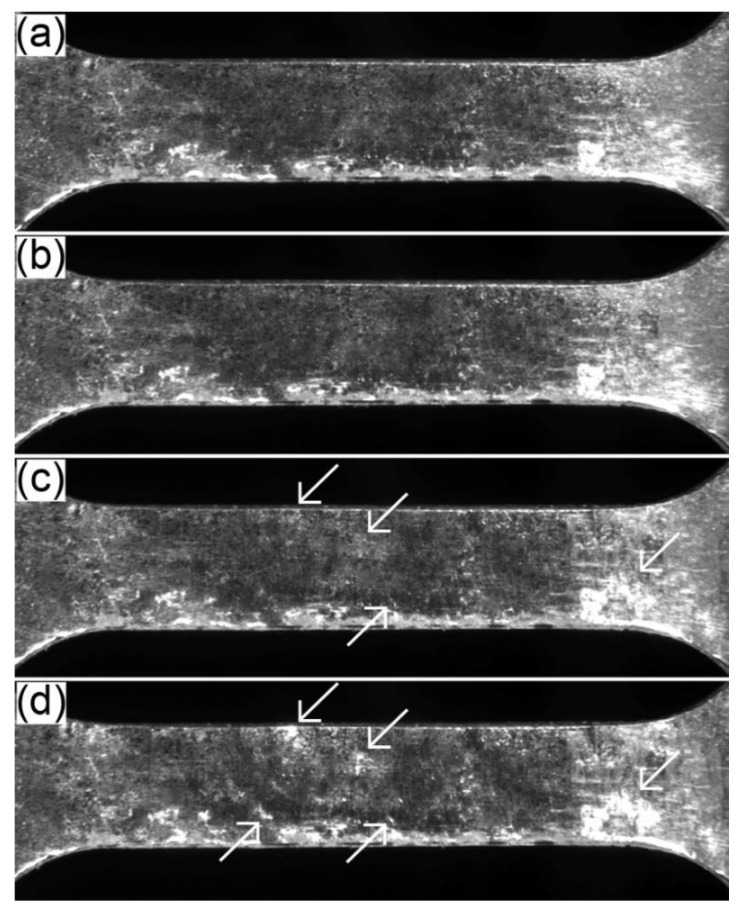
The changes tracked by the video recording system on the surface of the 4ZnCl_2__AC specimen in the following stages of the tensile test: (**a**) before test; (**b**) yield point; (**c**) 2% permanent deformation; and (**d**) beginning of necking.

**Figure 9:**
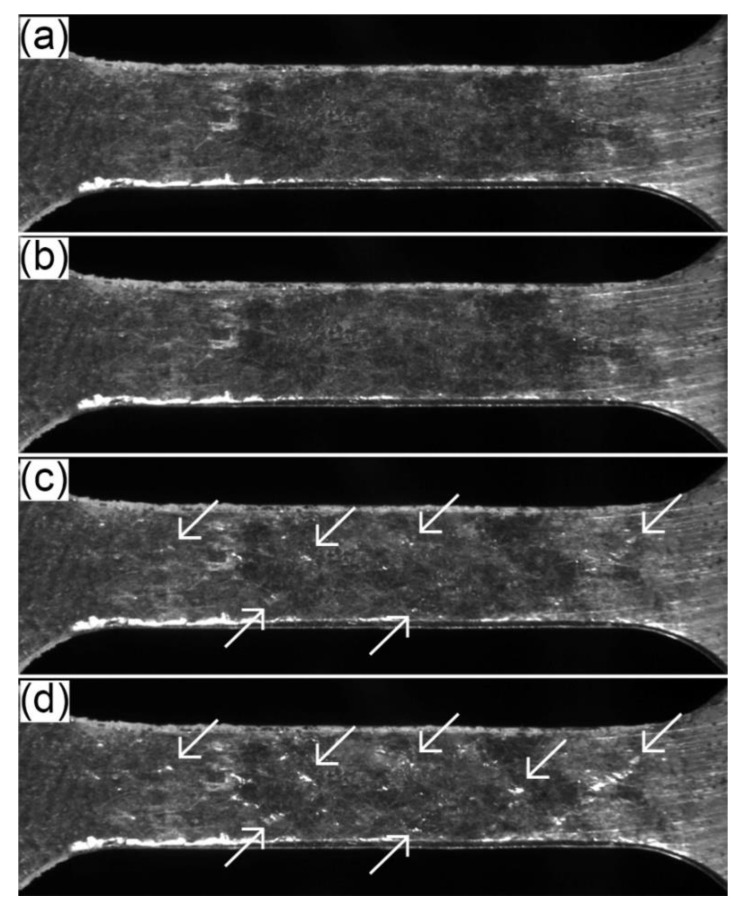
The changes tracked by the video recording system on the surface of the 4ZnCl_2__WC specimen in the following stages of the tensile test: (**a**) before test; (**b**) yield point; (**c**) 2% permanent deformation; and (**d**) beginning of necking.

Figure 8 shows the surface observed in the 4ZnCl_2__AC specimen during the tensile test, while Figure 9 illustrates the results for the 4ZnCl_2__WC variant. The images capture the following stages: before the test, at the yield point, at 2% permanent deformation, and at the beginning of necking. In both cases, the surface layer exhibited similar behavior. No damage to the layer was observed at the yield point. However, as plastic deformation progressed, the layer began to degrade locally in the form of chipping (visible as bright reflections in the images corresponding to 2% permanent deformation and the onset of necking).

**Figure 10:**
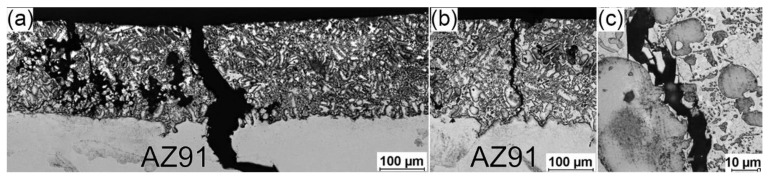
Optical micrograph of the surface layer in fractured 4ZnCl_2__AC specimen: (**a**) immediate vicinity of the local necking; (**b**) crack beyond the local necking; (**c**) high magnification of the crack.

**Figure 11:**
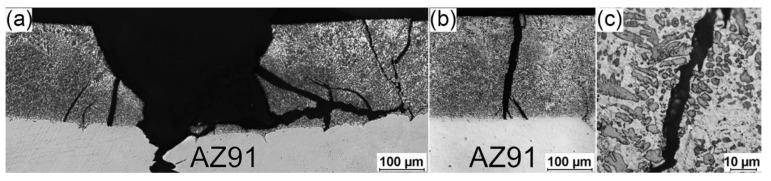
Optical micrograph of the surface layer in the fractured 4ZnCl_2__WC specimen: (**a**) immediate vicinity of the local necking; (**b**) crack beyond the local necking; and (**c**) high magnification of the crack.

Figure 10 and Figure 11 present the optical microscope examination of the fractured specimens for the 4ZnCl_2__AC and 4ZnCl_2__WC variants, respectively. The micrographs show that the cracking process was very similar in both cases. In the immediate vicinity of the local necking (Figure 10a and Figure 11a), brittle fracture across the surface layer resulted in the presence of numerous chippings. Outside the necking area (further from the fracture shown in Figure 10b and Figure 11b), single perpendicular brittle cracks through the layer and surface chipping were observed. Figure 10c and Figure 11c display high magnification micrographs of the fractures in the analyzed surface layers. The cracks tended to propagate through the region of the Mg_5_Al_2_Zn_2_ intermetallic phase, clearly avoiding the disruption of the dendrites of the Mg-solid solution. It was reported in the literature data (for instance, [25]) that the Mg-solid solution has superior mechanical properties compared to the Mg-Al-Zn intermetallic phases, which may explain this observed phenomenon. Fractographic analysis also confirmed that, in both variants, no delamination was observed at the interface between the surface layer and the AZ91 substrate.

## 4. Discussions

Heating the AZ91 alloy in contact with a ZnCl_2_-containing mixture resulted in the formation of a Zn-enriched layer on its surface. Moreover, by carefully selecting the medium composition, it was possible to control the thickness of the resulting surface layer. At ZnCl_2_ concentrations of 3% and 4%, the layers were relatively uniform, with thickness ranging from 40 µm to 200–240 µm, respectively, indicating that the layer thickness can be effectively controlled within this range. However, increasing the ZnCl_2_ concentration above this value resulted in the formation of layers with irregular thickness and numerous defects, making it difficult to achieve a high-quality layer with greater thickness.

The formation of the discussed surface layer indicates that a displacement reaction occurs between ZnCl_2_ and the surface of the Mg-based alloy during the process. Consequently, Zn atoms are deposited on the AZ91 surface, where they can diffuse into the substrate at elevated temperatures. The significant concentration of Zn at the alloy surface leads to the formation of low-melting phases, resulting in the formation of the surface layer through the involvement of a liquid phase. The final thickness and composition of the surface layer depend on the cooling rate and the content of the ZnCl_2_ in the mixture.

Age hardening is a multi-step heat treatment that involves heating the alloy to a specific temperature, followed by rapid cooling to maintain a supersaturated Mg-solid solution. The cooling rate is dependent on the material being processed, with water commonly used for this purpose. After cooling, the alloy is aged at either an elevated or room temperature, resulting in the formation of fine precipitates of intermetallic phases. The experimental results showed that air cooling was sufficient to supersaturate the AZ91 alloy. Consequently, subjecting AZ91 alloy to age hardening, whether cooled in air or water, led to an improvement in mechanical properties, with both variants yielding similar tensile strength characteristics. These results are consistent with the literature, which suggests that the supersaturation of Mg alloys can be achieved even at a relatively low cooling rate [21]. Furthermore, the complex treatment of the AZ91 alloy in contact with ZnCl_2_ also resulted in favorable mechanical properties. The tensile strength in both air-cooled and water-cooled specimens was comparable to that of traditional age hardening without Zn enrichment. However, the study demonstrated that the cooling rate significantly affected the microstructure of the surface layer formed on the AZ91 substrate, with a higher cooling rate leading to a substantial refinement of the microstructure. Fractographic analysis for both cases revealed no cracking of the produced layers until the onset of necking in the specimen. Brittle perpendicular cracks and chipping of the surface layer started to occur together with significant plastic deformations. Importantly, no delamination was observed at the interface between the layer and the AZ91 substrate, indicating strong metallurgical bonding.

It is known that the treatment of Mg-based alloys at high temperatures under atmospheric conditions may lead to notable oxidation of their surface. For this reason, protective atmospheres are often applied, especially at temperatures greater than 400 °C. It is worth noting that a potential advantage of the method analyzed in this study is that the surface modification of Mg-based products can be carried out simultaneously with their heat treatment. Additionally, the forming layer can protect the surface against oxidation at elevated temperatures. This innovative and complex treatment, with the use of inexpensive materials, appears to be economically viable. However, a potential weakness is that ZnCl_2_ is a hazardous substance. Therefore, implementing this technology requires appropriate safety regulations and effective waste management protocols, which can be adapted from other processes using liquid salts [26].

## 5. Conclusions

The present study demonstrated that the complex treatment process, which included age hardening and surface layer modification, significantly enhanced the surface characteristics and mechanical properties of the AZ91 alloy. The main outcomes are as follows:

(1) The thickness and uniformity of the zinc-enriched surface layer were directly influenced by the ZnCl_2_ content in the mixture. Specifically, a 3% ZnCl_2_ concentration produced a uniform surface layer approximately 40 µm thick; a 4% ZnCl_2_ concentration increased the layer thickness to 200–240 µm; a 5% ZnCl_2_ concentration resulted in a thicker but uneven layer, ranging from 100 to 440 µm, with defects such as pores and irregularities.
(2) Moreover, the cooling method was shown to play a critical role in the final microstructure of the surface layer: Air cooling led to a complex microstructure with coarse dendrites, the Mg_17_Al_12_ phase, and MgZn particles near the interface. Water cooling produced a finer, uniform microstructure with reduced dendritic growth.
(3) The mechanical properties of the treated AZ91 alloy were significantly improved, with strength levels comparable to those of the T6 temper. The treated surface layers exhibited no cracking under moderate deformation, and despite some defects appearing under greater plastic deformation, no delamination was observed between the surface layer and the substrate.

In conclusion, the complex treatment process allows for the precise tailoring of the AZ91 alloy’s surface layer in terms of thickness and microstructure by adjusting the ZnCl_2_ content and cooling methods. These enhancements suggest the potential for this treatment process in applications requiring high-performance materials. Future research will focus on further evaluating the corrosion resistance, tribological properties, and hardness testing of these surface layers to assess their broader applicability in the metal industry.

## Figures and Tables

**Table 1:** Process parameters used in the work with the corresponding symbolic designation (water cooling—WC; air cooling—AC).

Symbolic Designation	Mixing ZnCl_2_ with Filler (wt.%)	Age Hardening	Artificial Aging
AZ91_0	as-cast AZ91		
3ZnCl_2__AC	3% + filler	425 °C/24 h/AC	175 °C/16 h/AC
4ZnCl_2__AC	4% + filler	425 °C/24 h/AC	
5ZnCl_2__AC	5% + filler	425 °C/24 h/AC	
4ZnCl_2__WC	4% + filler	425 °C/24 h/WC	
T6_AC	without medium	425 °C/24 h/AC	175 °C/16 h/AC
T6_WC	without medium	425 °C/24 h/WC	

**Table 2:** Results of the quantitative EDS analysis at points marked in Figure 4 and corresponding phase determination (at. %).

Point	Mg	Zn	Al	Phases Determined by EDS Analysis
1	90.82	2.78	6.40	Mg-solid solution
2	59.23	22.33	18.44	Mg_5_Al_2_Zn_2_
3	90.98	2.55	6.47	Mg-solid solution
4	60.47	10.78	28.75	Mg_17_Al_12_ (Mg_17_(Al,Zn)_12_)
5	54.12	45.14	0.74	MgZn

**Table 3:** Average tensile strength test results of the analyzed specimens.

Variant Designation	Yield Strength	Ultimate Strength	Elongation
	MPa	MPa	%
AZ91_0	79 ± 2	139 ± 4	2.2 ± 0.2
T6_AC	95 ± 2	189 ± 4	3.3 ± 0.3
T6_WC	96 ± 3	188 ± 5	3.1 ± 0.3
4ZnCl_2__AC	93 ± 4	191 ± 8	3.6 ± 0.9
4ZnCl_2__WC	95 ± 4	185 ± 6	3.2 ± 0.5

## Data Availability

Data are contained within the article.
